# Physical and psychosocial work environmental risk factors of low-back pain: protocol for a 1 year prospective cohort study

**DOI:** 10.1186/s12891-019-2996-z

**Published:** 2019-12-27

**Authors:** Rúni Bláfoss, Per Aagaard, Lars Louis Andersen

**Affiliations:** 1 Musculoskeletal Disorders and Physical Workload, National Research Centre for the Working Environment, Lersø Parkalle 105, DK-2100 Copenhagen, Denmark; 2 Department of Sports Science and Clinical Biomechanics, Research Unit for Muscle Physiology and Biomechanics, University of Southern Denmark, DK-5000 Odense, Denmark; 3 Sport Sciences, Department of Health Science and Technology, Aalborg University, DK-9100 Aalborg, Denmark

**Keywords:** Musculoskeletal diseases, Musculoskeletal pain, Pain threshold, Workload, Occupational stress, Workplace, Psychology, Sociological factors

## Abstract

**Background:**

Musculoskeletal disorders, and in particular low-back pain (LBP), are common among blue collar workers. In the work environment, both physical- and psychosocial risk factors exist. Working in warehouses in Denmark involve large quantities of occupational lifting, high work pace and a low degree of influence at work. This study investigates both acute and long-term associations between physical- and psychosocial work environmental factors and risk of LBP in warehouse workers. The specific study aims are to investigate 1) exposure-response associations between quantity of occupational lifting and short-term (day-to-day) changes in LBP, 2) the influence of accumulated workdays and rest days during a working week on LBP, 3) long-term association between occupational lifting exposure and LBP when assessed over 1 year, and 4) the role of psychological and social factors on the above associations.

**Methods:**

The present study is designed as a 1-year prospective cohort study that will examine full-time warehouse workers from up to five retail chains in Denmark. Study aims 1 and 2 will be addressed using objective data based on company records with information on weight of all the goods handled by each warehouse worker during every single workday for 3 weeks. During this period, each worker will reply to text messages received before and after every workday (also on days off work) in which study participants will score their pain in the low back, bodily fatigue and perceived mental stress (scale 0–10). Long-term pain development is assessed using questionnaire surveys before and after 1 year. Further, pressure pain threshold (PPT) will be measured for selected trunk extensor muscles in approximately 50 workers using algometry along with measurements of maximal trunk extensor strength. Associations are modelled using linear mixed models with repeated measures between variables and LBP controlled for relevant confounders.

**Discussion:**

This study provides knowledge about the acute and long-term associations between physical- and psychosocial work environmental factors and LBP. The obtained data will have the potential to provide recommendations on improved design of the working week to minimize the risk of LBP among warehouse workers, and may potentially enable to identify a reasonable maximum lifting threshold per day (ton lifted/day).

## Background

Musculoskeletal disorders are the leading cause of physical disability and a major burden on individuals and societies, with Low-back pain pain (LBP) representing the most frequent musculoskeletal disorder [1–3]. Workers exposed to regular occupational lifting are in elevated risk of LBP [4–7] while additionally characterized by an increased risk of long-term sickness absence, early retirement and earlier death [8–13].

**Fig. 1:**
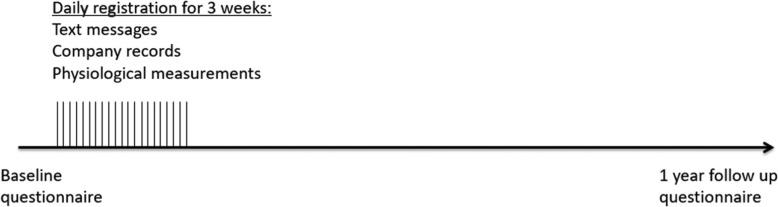
Time line of the study

**Fig. 2:**
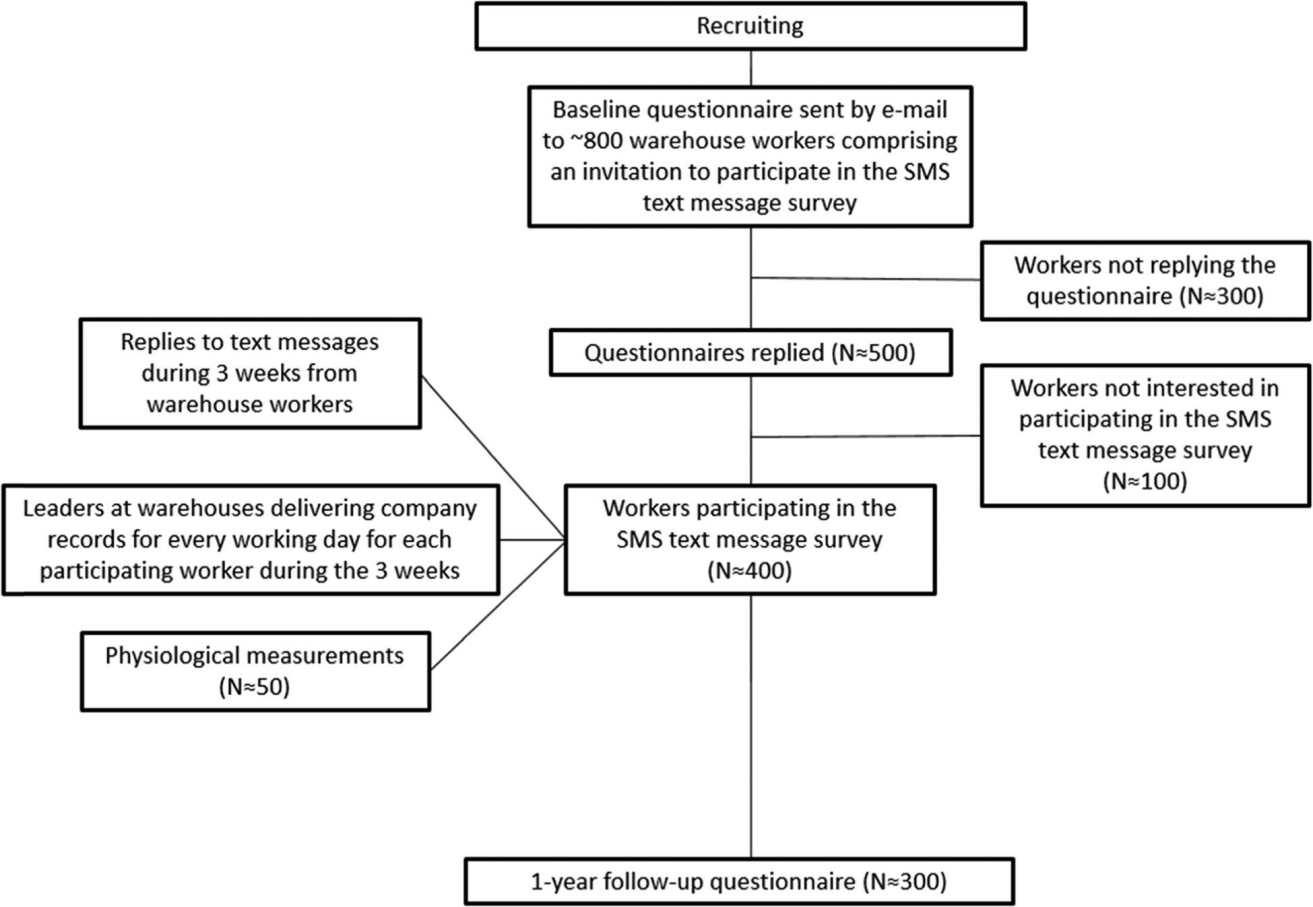
Flow chart of the study

Warehouse workers represent a job category with physically demanding job tasks that comprise large amounts of occupational lifting [14, 15]. In the 2018 Work Environment & Health study, warehouse- and transport workers in Denmark rated their job to be physically demanding with a mean score of 5.4 on a 0–10 scale [14]. The Work Environment & Health study evaluates both physical and psychosocial work environmental factors and overall health in a range of job types, with the latest survey including 38,000 respondents from the general working population in Denmark [14]. Besides rating their work as physically demanding, 36% of the warehouse- and transport workers reported musculoskeletal pain several times per week while 7% reported a limited work capacity due to pain [14]. Moreover, warehouse- and transport workers estimated that loads of 16 kg or more were lifted for about 26% of the workday. Thus, warehouse- and transport workers in Denmark are highly exposed to heavy occupational lifting, which per se is known to increase the risks of developing LBP [4–6, 16, 17]. Likewise, isolated warehouse work consists of both heavy and frequent lifting [14, 15]. Based on meta-analysis of the available literature, Coenen and co-workers investigated the influence of occupational lifting on the occurrence of LBP by quantifying duration, frequency and intensity of the lifting [6]. The study found odds ratios for LBP to be 1.11 per 10 kg lifted and 1.09 per 10 lifts per day [6]. In relation to this, Danish warehouse- and transport workers estimated their lifting burdens to be 16 kg or more for 26% of the workday [14]. Based on these odds ratios, Danish warehouse- and transport workers are exposed to heavy daily lifting loads (≥16 kg for 26% of the day) that may be expected to result in an elevated risk of LBP and related musculoskeletal disorders.

Musculoskeletal disorders, including LBP, are influenced not only by physical work factors, but also psychosocial work environmental factors, e.g. low job control and low social support [5, 17, 18]. Notably, Danish warehouse- and transport workers are scoring low on psychosocial work environmental factors [14], suggesting that Danish warehouse- and transport workers are exposed to a physically demanding job that typically comprise a poor psychosocial working environment.

Previous studies investigating risk factors for reduced health among workers with physically demanding job tasks typically have involved subjective measurements [5, 8–10, 19, 20]. However, a recent study among full-time supermarket workers investigated the association between occupational lifting and day-to-day variations in LBP [21]. Using internal company records to quantify the daily lifting volume and daily text message communication to assess pain intensity in the low back in 3 weeks, an exposure-response association was observed between workload intensity and LBP intensity, along with higher pain intensity the morning after workdays [21].

The aim of the present study, therefore, is to investigate the association between objectively assessed occupational lifting and perceived psychosocial work environmental factors and LBP intensity among warehouse workers in retail industry warehouses in Denmark. The objectives of the study is to investigate: 1) the exposure-response association between quantity of occupational lifting and short-term (day-to-day) changes in LBP, 2) the influence of accumulated workdays and rest days during a working week on LBP, 3) long-term association between occupational lifting exposure and LBP when assessed over 1 year, and 4) the role of psychological and social work environmental factors on the above associations. Our hypotheses is that higher total lifting loads will associate with more intense LBP, that LBP intensity increases during consecutive workdays and is higher at 1-year follow-up, and that these associations comprise psychological and social factors.

## Methods

### Study design and population

The present 1-year prospective cohort study investigates work-related symptoms among warehouse workers from up to five different retail chains industry warehouses in Denmark. Enrolled study participants will in February-March 2020 receive a baseline questionnaire by e-mail that will address various aspects of physical-, psychological and social work environment and health. The questionnaire will also comprise an invitation to participate in a text message survey. Participants recruited for the text message survey will receive a SMS text message before and after every workday for 3 full weeks (21 days) during March-April 2020 to rate the magnitude of pain in their low back, and to score their current level of perceived physical fatigue and mental stress. During the same 3-week study period, section leaders at the warehouses will provide company records about the workload of each participating warehouse worker (goods handled by each worker, weight of the goods) along with a working schedule for each worker. Additionally, during the same period, pressure pain threshold (PPT) will be measured in about 50 workers along with an optional assessment of maximal isometric trunk extensor strength (MVC) and systemic resting blood pressure. One year after responding to the baseline questionnaire, the participant will be receiving a follow-up questionnaire in February-March 2021 that will evaluate the physical-, psychological and social work factors and health aspects assessed 1 year previously. Fig. 1 illustrates a time line of the study.

Inclusion criteria for the warehouse workers recruited for this study are: working ≥30 h per week in a registered retail industry warehouse, ability to read and understand Danish or English, ≥18 years of age.

### Ethical aspects

According to Danish legislation, scientific questionnaire studies are not required to attain approval by official Danish ethical or scientific committees nor to obtain informed consent from study participants. Nevertheless, all questionnaires and text messages will be handled anonymously. A data manager at the research centre will store the data on a secure drive and de-identify the data before the researcher get access to these. All physiological measurements (PPT, MVC, systemic blood pressure) have been approved by The Danish National Committee on Biomedical Research Ethics (journal number: H-3-2010-062), and the project is registered at the Danish Data Protection Agency. Prior to giving their informed consent, all participants participating in the physiological measurements will be informed orally and in writing about the objectives and content of the study, as well as of their rights and potential risks of the study in accordance with the Helsinki Declaration.

### Baseline and follow-up questionnaires

The baseline and follow-up questionnaires contain questions about basic characteristics, general health and physical-, psychological and social work environmental factors. Questions about the physical work environment are based on previous investigations from our lab and the Work Environment & Health study [14, 21]. Questions pertaining the psychosocial working environment are selected from The Danish Psychosocial Work Environment Questionnaire (DPQ) [22] while questions about fear avoidance beliefs are selected from the Fear-Avoidance Beliefs Questionnaire [23]. The questionnaires will be sent by e-mail from a web-based survey platform (SurveyXact) while containing a web-link directed to the electronic questionnaire.

### Text message survey

All study participants will receive and reply to twice-daily SMS text messages before and after the workday for 21 consecutive days (also on days off work) [21]. The messages will be sent from a web-based survey platform (SurveyXact) containing a web-link that directs the participant to a 3-question survey about pain in low back, perceived physical fatigue and mental stress. To reply, participants choose a number between 0 and 10, where 0 is best and 10 is worst.

### Company records

During the 3-week observation period, warehouse section leaders will provide company records for all participants in the text message survey. These records will provide information on the specific goods handled (type and weight) manually by each participant during each single workday (workload), supplemented by a specific working schedule for each participant [21]. Based on these data the total weight lifted by each participant per workday for all 3 weeks will be calculated. Additionally, the repetitive lifting pattern of the different goods can be estimated from the amounts of each type of good and information about the total work duration, altogether allowing to quantify the duration, frequency and intensity of the physical work performed by each participant.

### Pain pressure threshold

During the 3-week observation period, PPT in the low-back muscles (m. erector spinae longissimus) is measured using an electronic pressure algometer (Somedic Productions AB, Sollentuna, Sweden, Europe) on approximately 50 participants for 2 days per week, i.e. comprising 6 measurement days in total. Control measurements will be conducted in a lower limb muscle (M. tibialis anterior) that is not directly affected by the manual lifting. The first PPT measurement is performed by the beginning of a working day preceded by a day off from work, while another PPT measurement is conducted during the working week just prior to initiating the fourth consecutive working day. PPT measurements are performed with a circular probe with a contact area of 1 cm^2^ at 3 contact sites on the m. erector spinae longissimus muscles on each side of the spine, and each contact area is measured 3 times with an interval time of 1½ minute [24, 25]. The test leader presses the algometer against the back muscles and slowly increases the pressure. Participants are informed to press a button on a pinch handle mounted to the algometer when the pressure changes from the feeling of pressure to the feeling of pain [24, 25]. The display of the pressure algometer will not be visual to the participants during the measurements. The PPT is expressed as the average value of the 3 measurements. All PPT measurements will be performed by the same test leader. Previous studies have found PPT both valid and a test-retest reliability level of satisfactory to good [26–31].

### Maximal isometric muscle strength

Participants in which PPT is determined will also be offered (encouraged) to have maximal voluntary contraction strength of the trunk extensors (MVC) assessed. Subsequent to the recording of PPT, MVC measurements are conducted at a working day preceded by a day off from work. Systemic blood pressure is measured prior to the MVC test and participants with blood pressure exceeding 160/100 mmHg will be excluded from testing [24, 32–34]. MVC measurements are performed using a standardized MVC procedure in a standing test-position in a custom-built device [34]. With a strap fixed around the shoulders, the back slightly flexed and leaning towards a pillow at the height of the anterior iliac spine, the participant will be informed to extend the back [34]. A warm-up trial consisting of 3 submaximal contraction efforts will be performed followed by 3 maximal MVC efforts separated by 1 min rest interval. Participants will be informed to slowly build up the contraction force to reach their maximum capacity after 2–3 s, and to continue the contraction until the test leader tells them to stop (approximately 3 s). Verbal encouragement will be provided throughout the test. The maximal isometric muscle strength will be expressed as the peak force produced during the 3 MVC trials.

### Statistical analysis

The baseline questionnaire will be sent out to ~ 800 warehouse workers (see Fig. 2 for flow chart illustration). Based on a previous study performed by our lab [21], we expect a participation and response rate of > 50% in the SMS text message survey, i.e. N ≈ 400. The section leaders at the warehouses have informed of a yearly worker turnover rate of ~ 14% in the different retail chains. Thus, when taken other factors into account, a drop-out rate of 20–30% is expected at the 1-year follow up, i.e. N ≈ 300. In 2017, Andersen & co-workers found significant day-to-day differences in LBP among 95 supermarket workers [21]. The sample size of the present study is therefore sufficient to investigate associations between risk factors at work and LBP.

The statistical analyses are based on methods previously published by our lab [21]. In brief, the statistical analysis will be based on linear mixed models with repeated measures (Proc Mixed, SAS version 9.4, SAS Institute, Cary, NC). LBP is the main outcome variable of the study and will be analysed as a continuous variable. PPT is a supplementary outcome and likewise treated as a continuous variable. Warehouses are entered as a random factor to account for clustering between work sites. Participant is entered as repeated factor using an autoregressive covariance structure. The estimation model is restricted maximum likelihood with degrees of freedom based on Satterthwaite approximation. The explanatory variables (fixed factors) for the first 3 objectives are 1) kg lifted per working day, 2) combination of working days and days off from work, and 3) mean of daily lifting (kg/day) during the 3 baseline weeks in relation to the change in LBP at 1-year follow-up. Analyses will be adjusted for relevant confounders as age, gender and psychosocial work environmental factors. For the fourth research question, the estimates of psychosocial variables will be drawn out of analyses 1–3, and we will also test whether the psychosocial factors interacts with the physical workload in relation to the change in LBP. Results are reported as least square mean differences with 95% confidence intervals (95% CI). An alpha-level below 0.05 is considered statistically significant.

## Discussion

The present cohort study will be the first to investigate acute- and long-term associations between physical and psychosocial work environmental factors and LBP among retail industry warehouse workers. The study will provide valuable knowledge about the risk of LBP and the day-to-day and weekly variations in pain intensity, while also investigating the development in LBP during a 1-year observation period in Danish retail industry warehouse workers. The obtained results potentially provide a basis to establish an improved design of the daily and weekly work load distribution pattern among warehouse workers, and may potentially allow to identify a maximum lifting threshold per day effective of reducing the risk of LBP in this large occupational group of workers.

### Strengths and limitations

A methodological strength of the present study is the objective quantification of individual day-to-day workload based on company records. These records provide precise data on the weight and quantity of each good handled by each employee. By contrast, the majority of previous research is based on self-reports of ergonomic exposures. Moreover, the repeated measure design of the 3-week text message period strengthens the study by eliminating recall bias. Another strength is the 1-year follow-up assessments, which provides knowledge about the long-term effects of occupational lifting not revealed by the acute day-to-day or weekly effects observed during the initial 3-week observation period. Furthermore, it is a methodological strength of the study that objective PPT measurements are combined with self-reported subjective pain scores. Finally, the present measurement of maximal trunk extensor strength enables to investigate the potential association between LBP pain and regional musculoskeletal function. These data may provide important knowledge about the influence of adequate physical capacity on prevention of LBP in physically demanding job settings.

A number of potential study limitations may also be listed. Firstly, the present study does not provide data on the specific biomechanical exposure during work, i.e. does not consider if the workers are working with back bent or twisted, arms above shoulder height etc., which are factors known to increase the risk of musculoskeletal injury independently of the magnitude of load lifted [5, 8]. However, it is plausible that these factors will be quite equal between different workers, as they all handle goods at a warehouse. Secondly, the company records provide precise information about the weight and number of goods handled by each worker, but it does not tell the magnitude of the horizontal and vertical displacement distances the worker is handling the goods. However, this factor is also likely to be quite equal between the workers of the present study. Thus, while the results may be generalizable to manual material handling of goods similar to that in a warehouse, the results in terms of a safe lifting threshold may not be generalizable to all types of blue-collar workers.

## Data Availability

Researchers interested in collaborating using the data from the study should contact the corresponding author and senior author.

## Abbreviations

LBP: Low-back pain
MVC: Maximal voluntary contraction strength of the trunk extensors
PPT: Pressure pain threshold
